# Prostate cancer classification using 3D deep learning and ultrasound video clips: a multicenter study

**DOI:** 10.3389/fonc.2025.1582035

**Published:** 2025-06-27

**Authors:** Wenjie Lou, Peizhe Chen, Chengyi Wu, Qinghua Liu, Lingyan Zhou, Maoliang Zhang, Jing Tu, Zhengbiao Hu, Cheng Lv, Jie Yang, Xiaoyang Qi, Xingbo Sun, Yanhong Du, Xueping Liu, Yuwang Zhou, Yuanzhen Liu, Chen Chen, Zhengping Wang, Jincao Yao, Kai Wang

**Affiliations:** ^1^ Department of Intervention, Affiliated Dongyang Hospital of Wenzhou Medical University, Dongyang, Zhejiang, China; ^2^ College of Optical Science and Engineering, Zhejiang University, Hangzhou, China; ^3^ School of Medicine, Zhejiang University, Hangzhou, China; ^4^ Department of Ultrasound, Quzhou Affiliated Hospital of Wenzhou Medical University, Quzhou People’s Hospital, Quzhou, China; ^5^ Ultrasound Imaging & Interventional Therapy, Zhejiang Cancer Hospital, Hangzhou, China; ^6^ Hangzhou Institute of Medicine (HIM), Chinese Academy of Sciences, Hangzhou, China; ^7^ Department of Ultrasound of the Affiliated Dongyang Hospital of Wenzhou Medical University, Dongyang, China; ^8^ Department of Ultrasound, Yiwu Tianxiang Medical Oriental Hospital, Yiwu, Zhejiang, China; ^9^ Taizhou Cancer Hospital, Taizhou, China; ^10^ Department of Diagnostic Key Laboratory of Head & Neck Cancer Translational Research of Zhejiang Province, Hangzhou, China; ^11^ Zhejiang Provincial Research Center for Cancer Intelligent Diagnosis and Molecular Technology, Hangzhou, China; ^12^ Taizhou Key Laboratory of Minimally Invasive Interventional Therapy & Artificial Intelligence, Taizhou, China

**Keywords:** prostate cancer, ultrasound, deep learning, I3D model, multicenter study

## Abstract

**Objective:**

This study aimed to evaluate the effectiveness of deep-learning models using transrectal ultrasound (TRUS) video clips in predicting prostate cancer.

**Methods:**

We manually segmented TRUS video clips from consecutive men who underwent examination with EsaoteMyLab™ Class C ultrasonic diagnostic machines between January 2021 and October 2022. The deep learning-inflated 3D ConvNet (I3D) model was internally validated using split-sample validation on the development set through cross-validation. The final performance was evaluated on two external test sets using geographic validation. We compared the results obtained from a ResNet 50 model, four ML models, and the diagnosis provided by five senior sonologists.

**Results:**

A total of 815 men (median age: 71 years; IQR: 67–77 years) were included. The development set comprised 552 men (median age: 71 years; IQR: 67–77 years), the internal test set included 93 men (median age: 71 years; IQR: 67–77 years), external test set 1 consisted of 96 men (median age: 70 years; IQR: 65–77 years), and external test set 2 had 74 men (median age: 72 years; IQR: 68–78 years). The I3D model achieved diagnostic classification AUCs greater than 0.86 in the internal test set as well as in the independent external test sets 1 and 2. Moreover, it demonstrated greater consistency in sensitivity, specificity, and accuracy compared to pathological diagnosis (kappa > 0.62, p < 0.05). It exhibited a statistically significant superior ability to classify and predict prostate cancer when compared to other AI models, and the diagnoses provided by sonologists (p<0.05).

**Conclusion:**

The I3D model, utilizing TRUS prostate video clips, proved to be valuable for classifying and predicting prostate cancer.

## 1 Introduction

Prostate Cancer (PCa) ranks as the second most common cancer among men globally, as reported by the World Health Organization (GLOBOCAN) database (1). Its incidence and mortality rates are increasing, posing a significant threat to the physical and mental health of the male population, and representing a major public health concern.

PCa is a serious and potentially life-threatening illness that often goes unnoticed until it has reached advanced stages. Early detection and diagnosis are key to successful treatment outcomes. Unfortunately, identifying the disease in its early stages can be quite difficult, as the symptoms tend to be mild or even non-existent. The primary methods for early PCa screening include measuring levels of prostate-specific antigen (PSA) in human serum, conducting digital rectal examinations, and performing transrectal ultrasound-guided prostate biopsies. Even with these screening methods, accurate diagnosis remains a challenge (2). Doctors often have to rely on their own judgment, which can be subjective and lead to inconsistent results. Furthermore, the physiological changes that come with aging can affect the accuracy of diagnosis and the course of treatment. Early identification of PCa is critical for successful treatment, and further development of more accurate and non-invasive diagnostic methods is crucial (3). With the incidence and mortality rates of prostate cancer on the rise, it is more important than ever to find ways to detect and diagnose the disease in its early stages. This will, hopefully, lead to more successful treatment outcomes and a better quality of life for those affected by PCa.

Recently, studies utilizing deep convolutional neural networks (DCNNs) based on magnetic resonance imaging (MRI) and pathological sections have been conducted in the field of prostate cancer (PCa) (4–8), highlighting their potential value in PCa detection and evaluation. TRUS, as an integral part of early screening, retains rich imaging and video data prior to biopsies, without imposing additional psychological and economic burden on patients. Recent studies (9) have demonstrated that combining TRUS with traditional machine learning (ML) models, such as support vector machines (SVM) or random forests (RF), can enhance diagnostic capabilities to a certain extent. However, the combination of TRUS video and 3D deep learning (DL) models for evaluating prostate tumors remains unexplored.

DCNNs, which employ techniques such as convolution, pooling, weight sharing, and network module stacking (10), have the ability to automatically infer and map underlying convolutional features, resulting in abstract high-level expression (4, 11, 12).

In this study, we employ the Inflated 3D ConvNet (I3D) approach on TRUS video clips. Unlike previously proposed 3D convolutional algorithms that require segmentation during training, I3D can process the entire video frame (13). It is anticipated that this approach will provide the most effective ultrasound classification model for prostate cancer. Additionally, to the best of our knowledge, this is the largest-scale study to date that combines transrectal ultrasound video and 3D DL models for the evaluation of prostate tumors.

## 2 Materials and methods

This retrospective multicenter study was approved by the local institutional review board, and informed consent was waived due to its retrospective nature. The study was conducted in compliance with national and international guidelines.

### 2.1 Study participants

**Figure 1:**
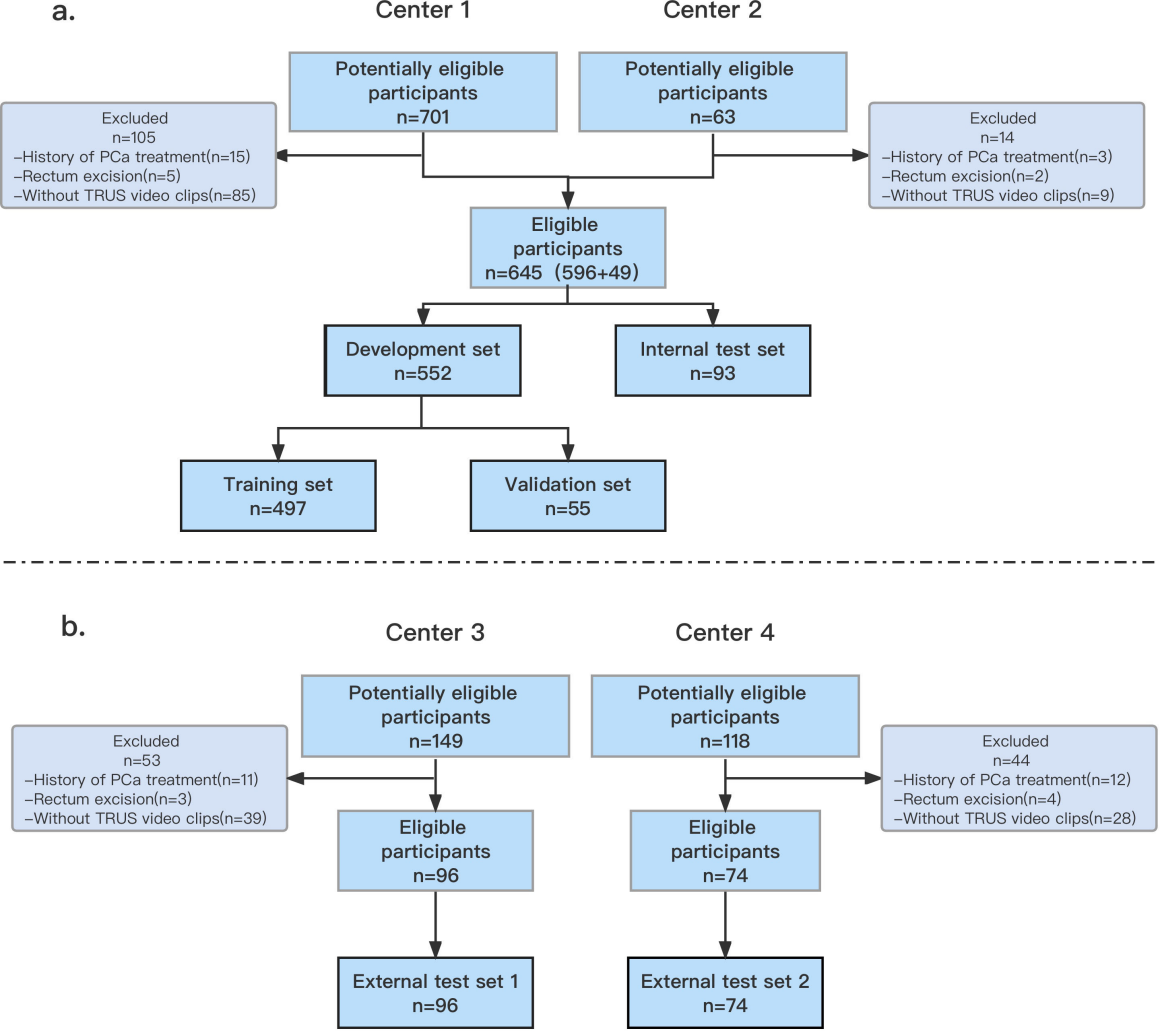
Flowchart **(a, b)** shows inclusion of patients into study. The data from Center 1 and Center 2 were randomly divided into a development set and an internal test set. The data from Center 3 and Center 4 were used as external test sets 1 and 2, respectively. PCa, prostate cancer; TRUS, transrectal ultrasonography.

Study participants comprised 1031 suspected prostate cancer cases, with TRUS video clips collected between January 2021 and October 2022. Among these, 701 cases were from Wenzhou Medical University Affiliated Dong yang Hospital (Center 1), 63 cases from Yiwu Tianxiang Medical Oriental Hospital (Center 2), 149 cases from China Medical University Cancer Hospital (Center 3), and 118 cases from The Quzhou Affiliated Hospital of Wenzhou Medical University (Center 4). All biopsy procedures across participating centers were performed under transrectal ultrasound (TRUS) guidance using a standardized 12-core systematic sampling protocol. No MRI-targeted biopsies were conducted in this study. The research enrolled patients who underwent TRUS-guided prostate biopsy at four medical institutions. Exclusion criteria were as follows: a) previous treatment for prostate cancer, such as radical prostatectomy, external-beam radiotherapy, brachytherapy, focal therapy, or androgen-deprivation therapy, as well as transurethral resection of the prostate or intravesical therapy; b) a history of rectal resection; c) a lack of retained prostate TRUS video data. For patients who had undergone multiple biopsy exams, only the results from the first biopsy examination were included in the analysis. Biopsy lesions were evaluated and classified based on the Gleason score. The enrollment of patients was conducted sequentially, as illustrated in **Figure 1**.

### 2.2 Collection of TRUS video clips

All TRUS video clips pertaining to the prostate were collected at our four medical centers using EsaoteMyLab™ Class C ultrasonic diagnostic machines (Esaote, Genoa, Italy) equipped with a TRT33 transrectal biplanar probe (frequency range 3–13 MHZ). The TRUS scans were performed by eight sonologists with more than five years of TRUS experience. The procedure followed the following criteria: Firstly, the sonologist explained the procedure to the patient, obtained informed consent for the prostate biopsy, reassured the patient, and ensured their understanding. Once confirmed, the patient was positioned on the examination bed in the supine lithotomy position. Secondly, the sonologist covered the TRT33 probe with a condom and inserted it into the rectum, adjusting the probe depth. They then manipulated the probe by pushing, pulling, and rotating it to facilitate a comprehensive view of the prostate from various directions. Finally, they scanned the entire cross-section of the prostate from top to bottom and saved video clips lasting five to ten seconds. Subsequently, a prostate biopsy was performed.

All original videos were acquired using the same model of ultrasound diagnostic equipment (Esaote MyLab™ Class C, Esaote, Genoa, Italy), with a native resolution of 608 × 800 pixels. To facilitate model training, we uniformly resized all frames to 256 × 256 pixels. Each video clip had a duration of 5–10 seconds, containing approximately 120–240 frames. However, since adjacent frames exhibited minimal differences, using all frames would have provided limited benefit for feature extraction while significantly increasing computational overhead. Moreover, the variable frame counts across different videos could have hindered model convergence. To address these issues, we adopted a uniform sampling strategy to standardize the input to 32 frames per video. This number was empirically determined based on our preliminary experiments. In practical applications, the sampling rate can be adjusted according to the original video length.

### 2.3 Manual segmentation and image registration

For manual segmentation, the 3D-slicer software (version 5.03) was utilized. The region of interest (ROI) in all video clips was selected as the entire prostrate, delineated by three sonologists, each with over five years of TRUS diagnosis experience. To ensure unbiased contouring, each case was anonymized by assigning a randomized number, thereby preventing the sonologists from accessing any relevant information prior to contouring. **Supplementary Figure E1** (available online) displays examples of manual segmentation. Subsequently, the sonologists marked and outlined the prostate shape, assigned positive/negative labels based on the actual pathological conditions of the patients, and identified video frames containing nodules for further processing. The dataset’s video clips contained additional information besides the patient’s prostate image. During data processing, irrelevant information regions were removed through clipping, preserving the original aspect ratio of the image. Additionally, the resolution of the video clips was scaled to 256x256.

### 2.4 Ultrasonic diagnosis

The diagnosis of PCa or non-PCa for all patients in the test sets was conducted by five sonologists each with over 15 years of experience in ultrasonic diagnosis. The sonologists assessed the ultrasound images and considered clinical data such as age, PSA levels, and other relevant factors. Throughout the diagnostic process, they were unaware of any information that could reveal the patient’s identity or the results of pathological diagnosis. The final ultrasonic diagnosis was determined through a voting system among the five sonologists, with the majority decision being followed.

### 2.5 Construct models

In this study, the I3D model was selected as the framework for classifying benign and malignant prostate lesions. The software used to develop the I3D model was based on the Ubuntu 18.04 operating system and included pytorch1.8.1 and Python (version 3.8). The training sessions were conducted on an Intel Core I7-7740X CPU operating at 4.30 GHz, paired with an NVIDIA GeForce TITAN Xp GPU. To extend the ConvNet-2D network to ConvNet-3D, a temporal dimension was added. ResNet 50 was employed to extract image features, which were then fed into the network’s output layer. The Softmax layer determined the network’s confidence in predicting benign and malignant nodules. The model utilized 32 frames of video sequences, with every other frame selected for processing. During training, random frames were selected, and data augmentation techniques were employed to prevent overfitting. The augmented data was fed into the pre-trained I3D model with a batch size of four. The model was trained with an initial learning rate of 0.0001, a decay rate of 0.000005 and optimized using AdamW with a weight decay of 0.001. The selected loss function was Cross Entropy.

To evaluate the performance of the I3D algorithm, it was compared with the DL ResNet 50-2D and ML algorithms. In the case of the ResNet 50-2d model, ResNet50 was utilized with a pre-trained model from the ImageNet dataset. Each frame of the video slice was treated as a separate input, with various enhancements such as flip, rotation, cropping, contrast, light adjustments, and Cutmix. The output was obtained through pooling and full link layers, and the results were averaged across all frames of each video sequence. The Softmax layer was employed for distinguishing between benign and malignant cases. In our study, we adopted a ResNet50-based I3D model as the backbone network. This architecture consists of approximately ​25 million trainable parameters, comprising multiple residual blocks with ​3D convolutional layers, batch normalization, and ReLU activation functions. By extending traditional 2D convolutional networks to the temporal dimension, our I3D model effectively captures ​both spatial and temporal information​ from video sequences.

The I3D algorithm followed the same training flow as mentioned above, and the parameters of the model with the highest accuracy were saved for testing. The Softmax layer was employed for benign and malignant discrimination. The training parameters and source code can be found online. https://github.com/NatsumeTetsuya/I3D-in-PCa-Classfication.

To evaluate the performance of the I3D algorithm, it was compared with ML algorithms. The pyradiomics library was used to extract features from prostate video data, which were filtered using the Lasoo regression algorithm and reduced to 17 dimensions (**Supplementary Figure E1**, available online). Four ML models (XGB, GBM, SVM, and RF) were trained using the selected features. Further details regarding the construction of the ML models’ construction can be found in **Appendix E2** (available online).

**Figure 2:**
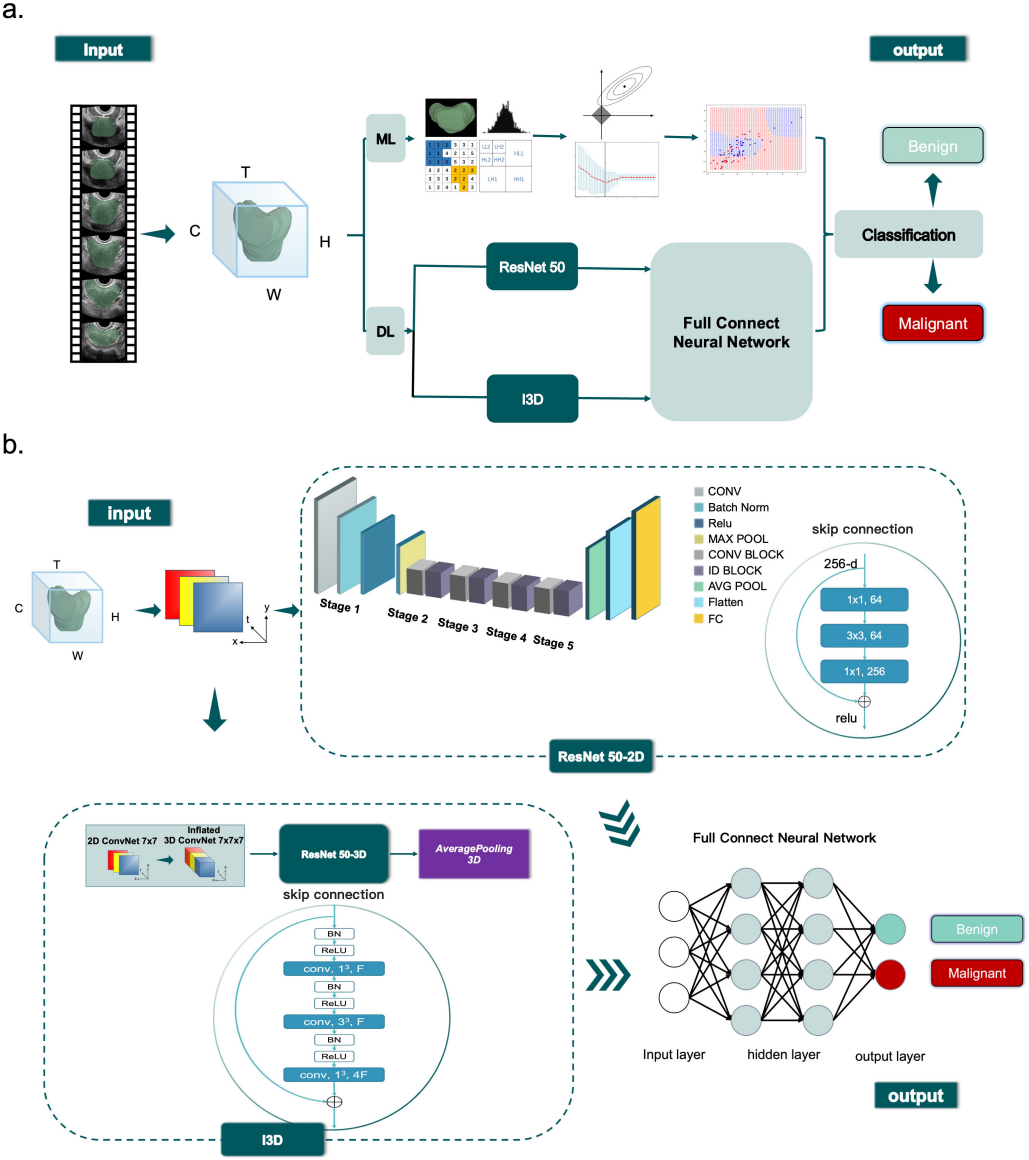
Diagram **(a)** shows overview of the ML (ML) and DL (DL) classification process. Diagram **(b)** shows ResNet 50 and I3D workflow.

The flowcharts of machine learning and deep learning are presented in **Figure 2**.

### 2.6 Statistical analysis

Normal distribution was assessed using probability-probability plots. Continuous variables were presented as mean ± SD, while categorical variables were presented as the number of patients and percentages. Paired sample t-tests were conducted to compare continuous data. The sensitivity, specificity, negative predictive value (NPV), and positive predictive value (PPV) of each cut-off point were compared using Chi-square tests or Fisher’s exact probability method. Agreement between the model and pathological diagnosis was measured using Kappa analysis. Model performance was evaluated and compared using AUC, and pairwise comparisons of AUC values were conducted to determine significant differences. Confidence intervals for ROC curves were estimated using the Delong test. Statistical significance was defined as p<0.05. Python and IBM SPSS Statistics 25.0 were utilized for statistical analyses.

## 3 Results

### 3.1 Baseline characteristics

Out of the 1031 men who presented to our institutions during the inclusion period, 815 (median age: 71 years; IQR: 67–77 years) met the inclusion and exclusion criteria. The study sample was randomly divided into different subsets: a development set (*n* = 552 [85%]; median age: 71 years; IQR: 67–77 years), an internal test set (*n* = 93 [15%]; median age: 71 years; IQR: 67–77 years), external test set 1 (*n* = 96; median age: 70 years; IQR: 65–77 years) and external test set 2 (*n* = 74; median age: 72 years; IQR: 68–78 years). **Table 1** provides demographic data and patient characteristics.

**Table 1:** Patient characteristics.

Characteristic	Development set (n=552)	Internal test set (n=93)	External test set 1 (n=96)	External test set 2 (n=74)
Median age (y)*	71 (67-77)	71 (67-77)	70 (65-77)	72 (68-78)
Median PSA (ng/mL)*	8.4 (5.5-28.0)	8.8 (6.1-17.1)	9.4 (5.8-28.0)	9.2 (5.4-27.0)
Benign subtypes	289 (52)	48 (52)	48 (50)	33 (45)
BPH	244 (44)	43 (46)	41 (43)	22 (30)
BPH & prostatitis	33 (6)	3 (3)	5 (5)	9 (12)
BPH & BCH	4 (0.7)	2 (2)	2 (2)	2 (3)
BPH & LGIN	8 (1.4)	0	0	0
Maximum Gleason score	263 (48)	45 (48)	48 (50)	41 (55)
GS6	111 (20)	15 (16)	11 (12)	12 (16)
GS7	94 (17)	17 (18)	18 (19)	10 (14)
GS8	37 (7)	6 (7)	13 (14)	16 (22)
GS9	17 (3)	6 (7)	6 (6)	3 (4)
GS10	4 (0.7)	1 (1)	0	0

For the training and validation sets, 90% and 10% of patients from the development set were included, respectively. The internal test set was randomly selected from Center 1. External test sets 1 and 2 were obtained from two other centers to provide geographic validation. Malignant lesions included Gleason scores 6-10, while benign lesions included benign prostatic hyperplasia (BPH), prostatitis, basal cell hyperplasia (BCH), and low-grade intraepithelial neoplasia (LGIN).

*Data in parentheses are the interquartile range.

In the internal training set, there were 48 (48/93, 52%) cases of benign prostatic lesions and 45 (45/93, 48%) cases of prostate cancer. The external test set 1 consisted of 48 (48/96, 50%) cases of benign prostatic lesions and 48 (48/96, 50%) cases of prostate cancer. In the external test set 2, there were 33 (33/74, 45%) patients with benign prostate lesions and 41 (41/74, 55%) patients with prostate cancer. The benign prostatic lesions included benign prostatic hyperplasia (BPH), prostatitis, basal cell hyperplasia (BCH), and low-grade intraepithelial neoplasia (LGIN), PCa was classified into 6 to 10 points based on the Gleason score.

### 3.2 Model performance on the internal test set and comparison with other models

In the internal test set, the I3D model demonstrated an AUC of.91, sensitivity of 91% (41/45, 95%CI: 78%, 97%), specificity of 85% (41/48, 95%CI: 72%, 94%), and overall accuracy of 88% (82/93, 95%CI: 80%, 93%). The agreement between I3D model assessment and ground truth, as measured by the Kappa value, was.76. The F1 score for classifying the prostate lesions as malignant or benign was 0.88.

Compared to Resnet 50 and the best ML model (SVM), the I3D model performed significantly better (AUC of.91 vs.75 and.82). It also outperformed the diagnosis of the sonographers (AUC of.60). The p-value of the Delong test was less than 0.05, indicating statistical significance.

### 3.3 Model performance on the external test set and comparison with other models

In the external test set 1, the I3D model achieved an AUC of.87, with a sensitivity, specificity, and accuracy of 83% (40/48, 95%CI: 69%, 92%); In test set 2, the AUC was.86, with a sensitivity of 81% (33/41, 95%CI: 64%, 92%), specificity of 82% (27/33, 95%CI: 64%, 92%), and accuracy of 81% (60/74, 95%CI: 71%, 88%). The agreement between the I3D model assessment and ground truth, as measured by the Kappa values, was 0.67 and 0.62, respectively. The F1 scores for classifying prostate lesions as malignant or benign were both 0.83.

In both external test sets, the I3D model outperformed Resnet 50 and the best ML model (GBM) (AUC: 0.87 vs 0.75 and 0.82; 0.86 vs 0.71 and 0.66). The p-values of the Delong test were less than 0.05, indicating statistical significance. The I3D model also performed significantly better than the diagnosis of the sonographers (AUC: 0.61 and 0.61). The p-values of the Delong test were less than 0.05, demonstrating statistical significance. Detailed information on the Delong can be found in **Appendix E3** and **Supplementary Table E1**.

**Table 2** presents the classification performance of the best ML model, ResNet 50 model, I3D model, and ultrasound doctors’ diagnosis in the test sets.

**Table 2:** Performance of models for classification of PCa in the test sets.

Sets and Models	Sensitivity [95%CI](%)	Specificity [95%CI] (%)	Accuracy [95%CI] (%)	F1 score	kappa
Internal test					
SVM	80(36/45)[65, 90]	75(36/48)[60, 86]	77((72/93)[68, 85]	.77	.55
ResNet50	47(21/45)[32, 62]	**94(45/48)[82, 98]**	71(66/93)[61, 79]	.61	.41
I3d	**91(41/45)[78, 97]**	85(41/48)[72, 94]	**88(82/93)[80, 93]**	**.88**	**.76**
Doctors	71(32/45)[55, 83]	48(23/48)[34, 63]	59(55/93)[49, 69]		.19
External test 1					
GBM	**92(44/48)[79, 97]**	56(27/48)[41, 70]	74(71/96)[64, 82]	.78	.48
ResNet50	52(25/48)[37, 67]	81(39/48)[67, 91]	67(64/96)[57, 75]	.61	.33
I3d	83(40/48)[69, 92]	**83(40/48)[69, 92]**	**83(80/96)[69, 92]**	**.83**	**.67**
Doctors	67(32/48)[51, 79]	56(27/48)[41, 70]	61(59/96)[51, 71]		.23
External test 2					
GBM	51(21/41)[35, 67]	**88(29/33)[71, 96]**	68(50/74)[56, 77]	.64	.37
ResNet50	63(26/41)[47, 77]	76(25/33)[57, 88]	69(51/74)[58, 78]	.69	.38
I3d	**81(33/41)[65, 91]**	82(27/33)[64, 92]	**81(60/74)[71, 88]**	**.83**	**.62**
Doctors	61(25/41)[45, 75]	61(20/33)[42, 77]	61(45/74)[49, 71]		.21

The data for sensitivity, specificity, and accuracy are expressed as percentages, with the corresponding proportions indicated in parentheses. All data enclosed in brackets represent 95% CIs. The highest evaluation metric values of models across all test sets are highlighted in bold in the table.

Further details are available in **Supplementary Table E2**.

**Figure 3:**
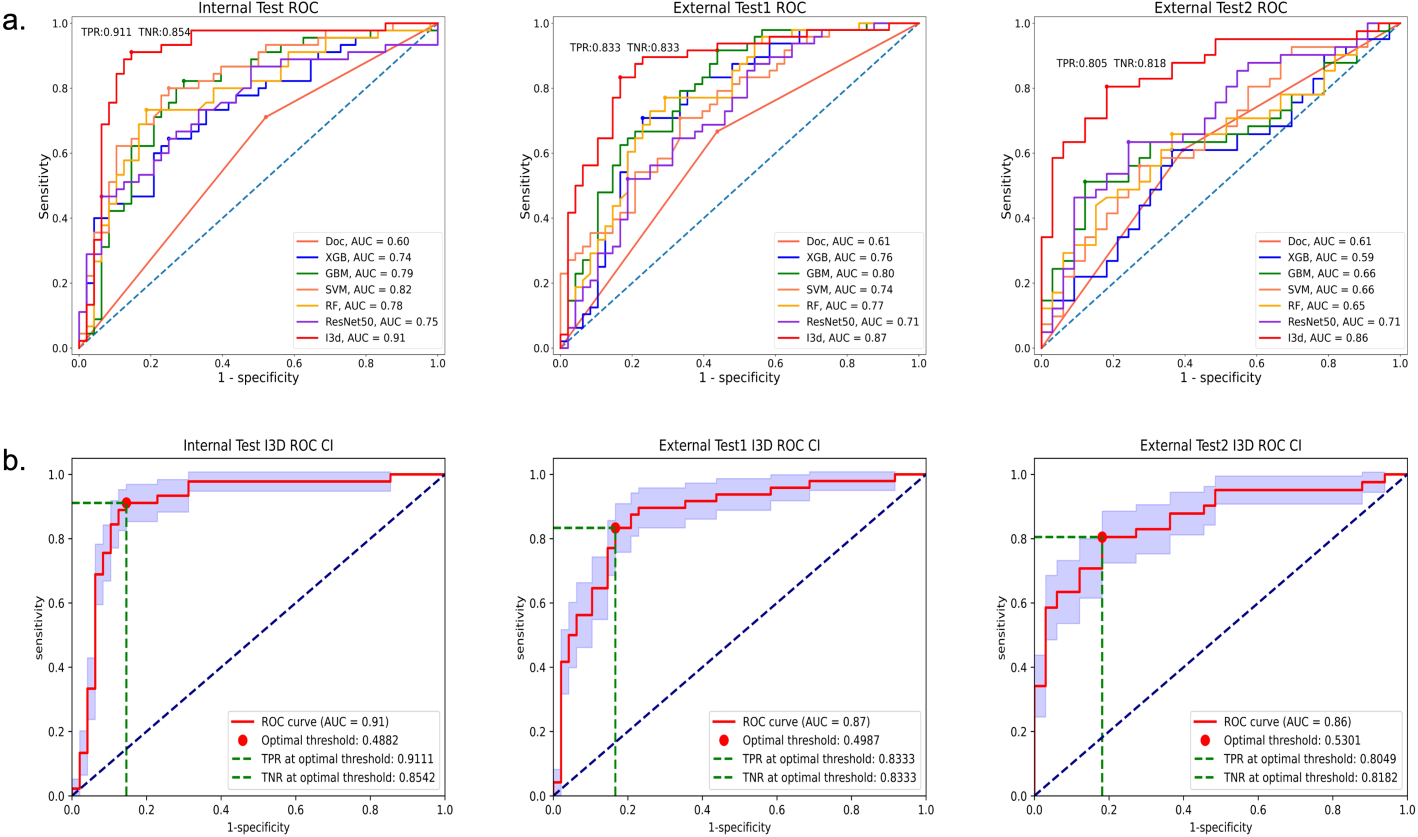
ROC of all models in the internal test, External test 1, and External test 2 sets. Graph **(a)** shows areas under the receiver operating characteristic (ROC) curve; the AUC values of the I3D model were superior to those of the other models. Graph **(b)** shows the ROC curve (95%CI, blue area), the optimal threshold, True Positive Rate (TPR) and True Negative Rate (TNR) of the I3D model in the three test sets.

**Figure 3** displays the ROC curves of all models in the internal test.

**Figure 4:**
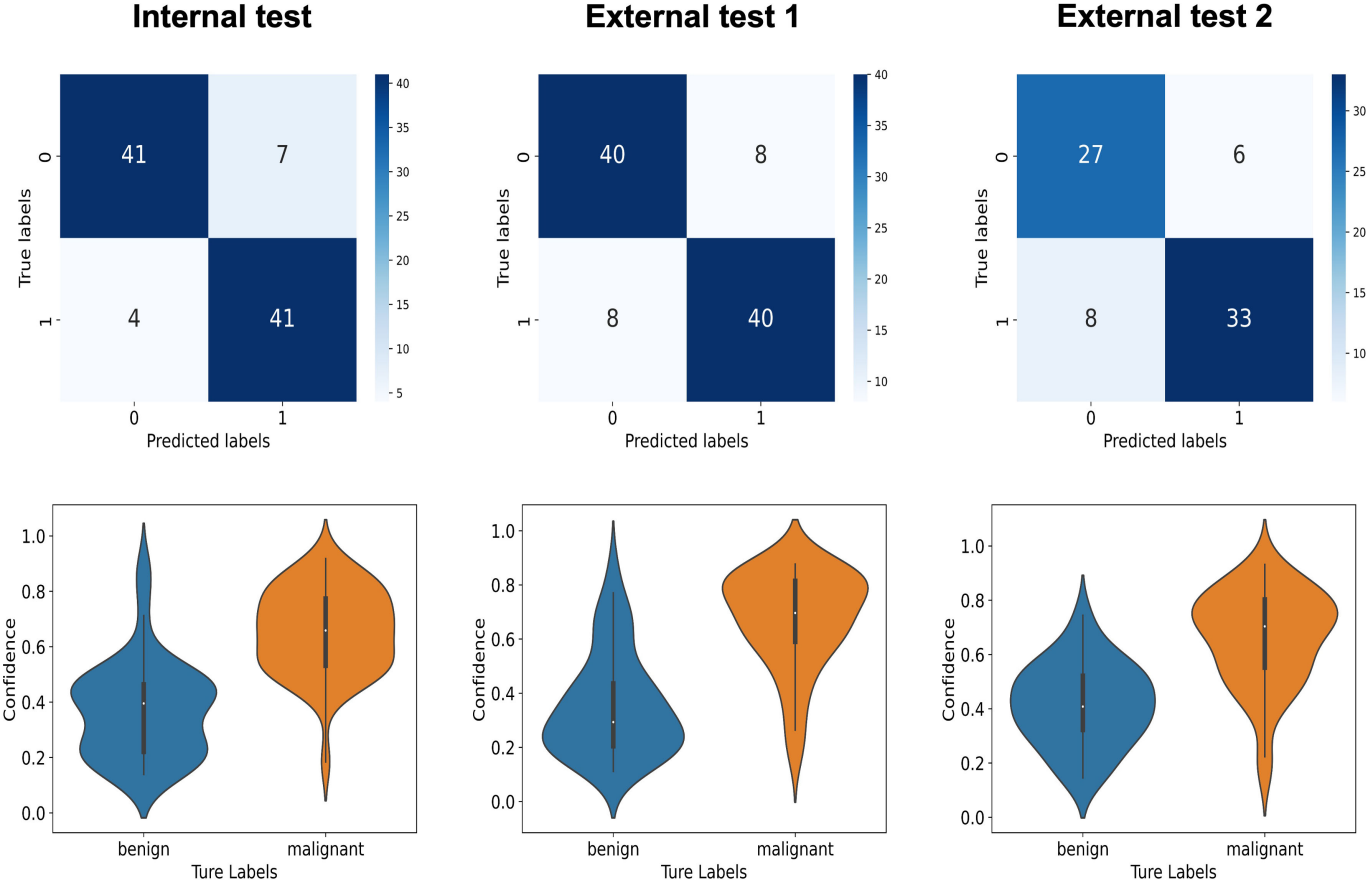
The upper graphs display the confusion matrices, while the lower graphs show the violin plots for the I3D model’s classification of benign and malignant tumors in the internal test set, the external test set 1, and the external test set 2.

**Figure 4** shows the confusion matrices and violin plots used for the I3D model to discriminate between benign and malignant tumors in the test set.

The confusion matrices and violin plots used for other models can be found in **Supplementary Figures E3**, **E4**.

**Figure 5:**
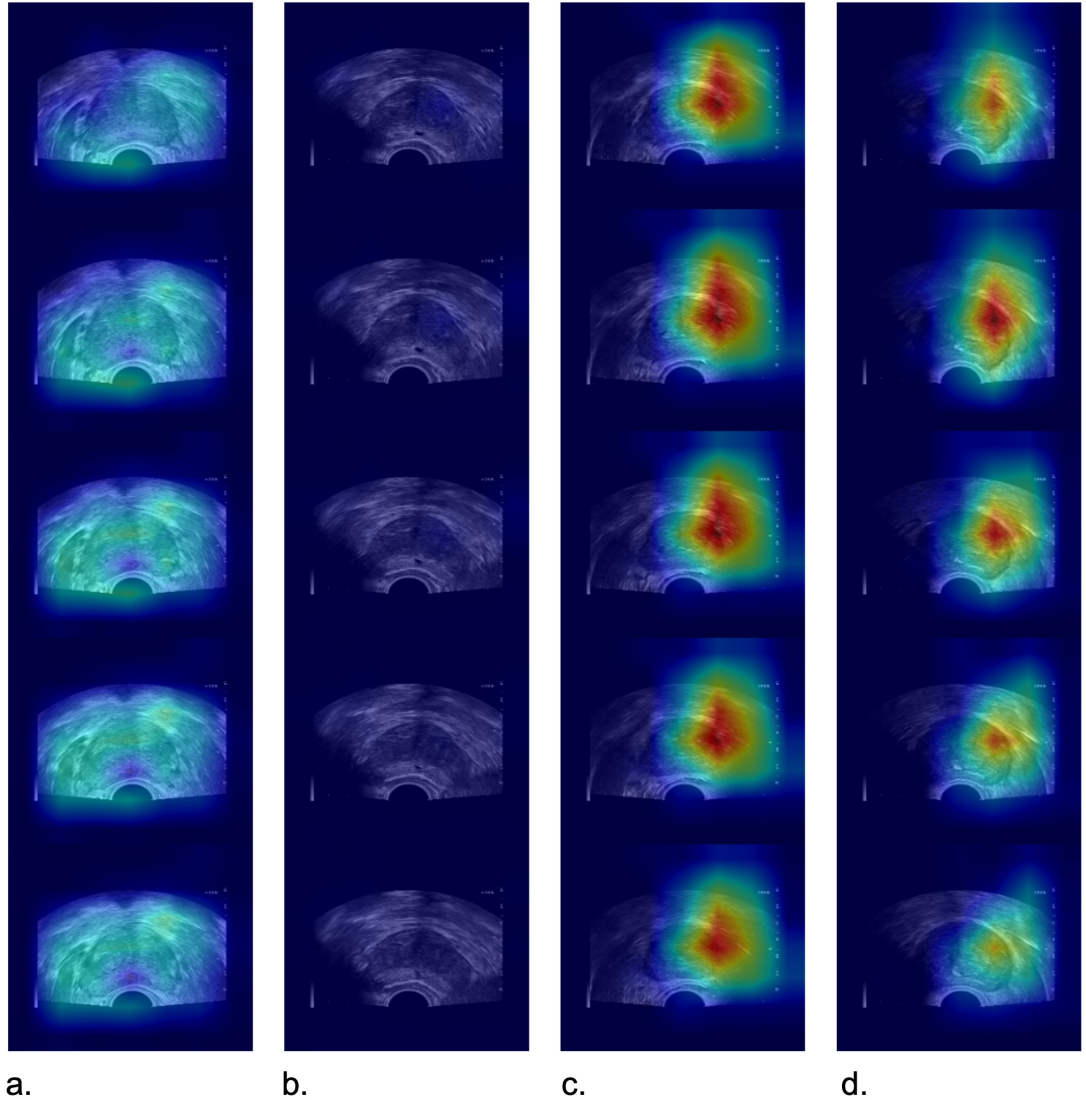
Images depict heatmap examples from TRUS videos of four patients in the external test set 2. In positive cases (c, d), the I3D model exhibited a relatively focused heatmap indicating the presence of prostate cancer. However, in negative cases (a, b), the attention was more diffuse, and there was no distinct focus area observed. **(a)** a 66-year-old man with a prostate-specific antigen level of 4.52 ng/mL and a biopsy pathology result indicating benign prostatic hyperplasia, **(b)** a 70-year-old man with a prostate-specific antigen level of 6.56 ng/m and a biopsy pathology result indicating benign prostatic hyperplasia, **(c)** a 74-year-old man with prostate-specific antigen level of 6.8 ng/mL and a biopsy pathology result indicating Gleason grade group 7, **(d)** a 78-year-old man with a prostate-specific antigen level of 17.81 ng/mL and a biopsy pathology result indicating Gleason grade group 6.

**Figure 5** displays heatmap examples derived from TRUS videos of four patients in External Test Set 2.

### 3.4 mpMRI-Negative Cases of PCa

For patients with suspected prostate cancer, we recommend undergoing an mpMRI examination before prostate biopsy. We reviewed a total of 90 MRI results from the internal test set (88/94), 70 results from the external test set 1 (73/96), and 50 results from the external test set 2 (52/74). mpMRI were acquired with 3.0T scanners (Siemens Magnetom Vida, Siemens, Erlangen, Germany, and Ingenia CX, Philips Healthcare, Best, Netherlands.). The mpMRI examination sequence includes T1WI, T2WI, DWI, ADC, and DCE-MRI.

There were 4 cases in our test sets with no lesions detected on prostate mpMRI, one case (GS6) is from the internal test set, while two cases (GS6, 7) are from the external test set 1, and one case (GS6) are from the external test set 2. However, not all patients may be willing to undergo an MRI examination, and we respect the patient’s preferences and wishes. Among them, three cases were diagnosed as prostate cancer by the I3D model, while one case was diagnosed as a benign lesion. **Table 3** shows the details of MRI-Negative Cases of PCa.

**Table 3:** The details of mpMRI-negative cases of PCa.

Cases	Group	Age	PSA	Pathology	mpMRI	I3D
1	Internal test set	79	16.24	Right peripheral zone with prostate adenocarcinoma, (Gleason score: 3 + 3 = 6, WHO/ISUP grade group 1).	BPH	B
2	External test set 1	83	6.83	Right central zone and right peripheral zone with prostate adenocarcinoma (Gleason score: 3 + 4 = 7, WHO/ISUP grade group 2).	BPH	M
3	External test set 1	68	14.38	Left central zone with prostate adeno-carcinoma (Gleason score 3 + 3 = 6, WHO/ISUP grade group 1).	BPH	M
4	External test set 2	84	3.48	Right peripheral zone prostate adeno-carcinoma (Gleason score: 3 + 3 = 6, WHO/ISUP grade group 1)	BPH	M

The pathological results of biopsy for case 3 showed a Gleason score of 7, while the results for other cases showed a Gleason score of 6. The mpMRI diagnosis did not find any obvious masses, only indicating benign prostatic hyperplasia (BPH). The diagnosis of I3D models for case 3 showed benign (B) and the diagnoses for other cases showed malignant (M).

## 4 Discussion

In this retrospective, multicenter, observational cohort study of 851 patients, we found that the I3D model based on TRUS video clips had superior diagnostic performance compared to the Resnet DL model, ML models, and experienced sonographers in classifying PCa. It demonstrated high accuracy even in cases where no obvious masses were detected on mpMRI examinations. These findings suggest that the I3D model can serve as a reliable predictive model for diagnosing PCa.

The selection of participants in a study is a crucial determinant of its representativeness and applicability. Our study identified patients who had undergone TRUS-guided biopsy as the inclusion criteria. This comprised individuals with elevated PSA levels, benign prostatic hyperplasia necessitating pre-operative pathology and suspicious nodules identified by MRI (14–16). By broadening the inclusion criteria, this study’s results have become more representative and applicable compared to previous investigations that only included patients with nodules diagnosed by MRI. Our test sets yielded a fascinating finding whereby despite four PCa cases with no tumor detected by MRI, the I3D model correctly diagnosed three individuals. Together with the broadening of the inclusion criteria, this has enhanced the representativeness and generalizability of this study’s findings. It is important to emphasize that the PI-RADS scoring system for mpMRI is inherently a probabilistic tool rather than a definitive diagnostic standard. The value of our model does not lie in judging the ‘accuracy’ of MRI but rather in ​​providing additional reference for PI-RADS category 3 cases, and​ serving as a complementary test for clinically suspicious yet MRI-negative cases.​​ This multiparametric decision-making approach aligns more closely with the principles of modern precision medicine. Although the model detected 3 out of 4 MRI-negative cancers, the limited sample size means this result requires validation in prospective large-scale studies. We recommend that future research incorporate multiple parameters such as PSA density and clinical indicators to establish a combined predictive model.

In our study, we employed prostate TRUS videos to create a deep learning model that improved the model’s accuracy and precision. Instead of relying only on images and delineating nodules (17, 18), we delineated all frames of the videos to generate mask files for the prostate tissue. By using video clips and delineating each frame comprehensively, we were able to evaluate prostate tissue characteristics more comprehensively. Consequently, these methodological innovations have rendered this study more reliable and innovative, offering novel insights and approaches in the field of PCa diagnosis. Compared with the MRI deep learning model (AUC 0.832) reported by Liu Zheng et al. (19), our TRUS video model achieved comparable performance. The advantages of TRUS lie in its high accessibility, low cost, and no need for additional examinations, making it particularly suitable for primary screening. However, it must be emphasized that MRI still holds irreplaceable value in localization and staging, and the two modalities should be considered complementary rather than competitive. A study conducted by Sun et al. utilized 832 prostate TRUS videos to construct a 3D convolutional neural network model (20). In terms of predicting clinically significant PCa, the internal validation cohort achieved an AUC of 0.89, sensitivity of 0.63, and specificity of 0.94, the external validation cohort achieved an AUC of 0.85, sensitivity of 0.81, and specificity of 0.78. However, their model incorporated clinical parameters (total PSA, free PSA, PAS density, family history, and previous negative biopsies) to enhance diagnostic performance. They trained a logistic regression classifier using the output probability of imaging predictors based on key clinical parameters and the 2D P-Net and 3D P-Net models, resulting in a clinical nomogram. In contrast, our model is solely based on TRUS video data and its diagnostic performance in predicting PCa is shown in **Table 2**.

In this study, we used a 3D DL model to analyze TRUS prostate videos and differentiate between benign and malignant prostate tumors. To our knowledge, this is the first study to combine ultrasound videos with 3D DL models to differentiate prostate tumors. In order to better compare the accuracy of different methods for TRUS, we compared the identification results of 3D DL model, traditional ML models, and radiologists. Among them, we used the I3d model for 3D DL (13). And it extend the 2D convolutional layers to 3D, allowing for the capture of both temporal and spatial information in videos (21–23). The results demonstrate high performance in diagnosing PCa using the I3D model, as evidenced by its sensitivity, specificity, and AUC. These findings support the hypothesis that the 3D DL model can extract valuable diagnostic information from TRUS prostate video clips. Furthermore, the evaluation of the 3D DL model is based solely on TRUS video clip data and has shown comparable performance to multiparametric MRI in previous studies (24–26).

ML and DL algorithms serve as powerful tools for analyzing the vast amount of available image data. They allow us to uncover complex underlying biological mechanisms and have the potential to enable personalized precision cancer diagnosis and treatment planning. These algorithms have demonstrated comparable accuracy to human experts (27–29), or have reduced interobserver variability (30, 31), or physician workload in various applications (32), including disease classification, image segmentation, outcome prediction, automatic treatment planning, motion trajectories, and image enhancement.

Our study has several limitations that should be acknowledged: a) The TRUS video clips utilized in our study were collected exclusively from ultrasound instruments of the same brand across the four centers. In future research, we will consider incorporating video clips captured by assorted brands of ultrasound instruments. b) Currently, our research is focused solely on gray-scale ultrasound image data. To further improve the classification performance of PCa, we intend to explore the integration of multi-modal data, such as contrast-enhanced ultrasound, clinical information, and MRI in constructing a DCNN model. c) DL modeling typically involves a black-box development process, where the algorithm learns from vast amounts of data points and produces outputs by associating specific data features. The process is largely self-directed by AI and can be challenging for data scientists, programmers, and users to interpret. d) The use of biopsy results as the gold standard in this study has certain limitations. Due to the sampling constraints of biopsies (particularly for MRI-negative lesions), false-negative results may occur. To mitigate this potential bias, all included cases underwent a standardized 12-core systematic biopsy protocol. For clinically suspicious cases with initial negative biopsies (e.g., those with persistently elevated PSA levels or positive MRI findings), repeat biopsies were recommended. Nevertheless, this limitation may affect the accuracy of model evaluation. In our future work, we aim to expand the dataset through collaboration with multiple centers to validate the generalizability of our model across diverse providers and patient populations. Additionally, we plan to strengthen our findings by conducting prospective studies.

## 5 Conclusions

Our proposed DL I3D model demonstrates promising feasibility in predicting PCa. Compared to the diagnosis of sonographers and ML models utilizing individual feature groups, the DL model based on TRUS prostate video clips significantly improves predictive classification performance. The proposed model has the potential to aid in identifying patients at higher risk of PCa and may contribute to reducing the number of unnecessary prostate biopsies.

## Data Availability

The original contributions presented in the study are included in the article/**Supplementary Material**. Further inquiries can be directed to the corresponding author.
