# Supplementary material for: Prostate cancer classification using 3D deep learning and ultrasound video clips: a multicenter study
Source: Front Oncol. 2025 Jun 27;15:1582035. doi: 10.3389/fonc.2025.1582035 (PMC12245699; doi:10.3389/fonc.2025.1582035)
Supplement: Supplementary file 3 [file DataSheet3.pdf]

Appendix

E1 Machine Learning (ML) Architecture

Four Machine Learning(ML) models were selected for training and testing, and compared with the I3d model in the benign and malignant classficaiton performance of TRUS prostate video slices, including Support Vector Machine (SVM), Random Forest (RF), XGBoost (XGB), and LightGBM (GBM) models. The training set, validation set, and test set for ML models were consistent with the dataset of I3d networks. 853-dimensional high-throughput features were extracted from video slice through the pyradiomics library. The features extracted from the training and validation sets were were filtered by the Lasoo regression algorithm and reduced to 17 dimensions(Figure E2[online]) using 10 fold cross validation. The filtered features were input into four ML models for training and validation, with fixed and searchable parameters set for each model. In training stage, the optimal parameters were determined by 8-fold cross validation(SVM:{'C':2.154, 'gamma':018}; RF:{'n_estimators':1000}; XGB:{'learningrate':0.1, 'max_depth':10, 'n_estimators':50}; GBM:{'n_estimators':20, 'num_leaves':31, 'max_depth':-1, 'min_data-in-leave':50, 'learningrate':0.1, 'subsample':0.7}). Test set were input into four trained models to obtain test results.

E2 Python Packages

Python3.8.10 was used as a programming language for data preparation, model training and results analysis. Specific libraries were used: numpy 1.24.1, torch 1.10.1, torchvision 0.11.2, mmaction 0.5.0, mmcv 2.0.1, albumentations 1.1.0, pandas 1.4.2, scikit-learn 1.0.2, scipy 1.8.0, matplotlib 3.5.2, tqdm 4.65.0, pynrrd 1.0.0, pyradiomics 1.0.0.

E3 Delong Test

1. Calculate the predicted probability values of each model at each sample point.
2. For each sample point, take the paired differences between the predicted probability values of the two models. That is, for a sample point, calculate the predicted probability of Model 1 minus the predicted probability of Model 2.
3. Sort the paired differences and calculate the difference in the area under the ROC curve (AUC) based on the sorted results.
4. Calculate the standard error (SE) of the AUC difference.
5. Calculate the z-value of the AUC difference, i.e., the AUC difference divided by the standard error.
6. Calculate the p-value based on the z-value and degrees of freedom (i.e., the number of sample points).

Table E1 Comparison of AUCs between the I3D Model and Other Models in Test Sets

Comparison	AUC	Standard error	Z_score	P_value	Z_alpha 95%CI
Internal test					
I3D vs Resnet 50	0.91 vs 0.75	0.02	7.55	4.29E-14	0.12, 0.20
I3D vs SVM	0.91 vs 0.82	0.02	4.5	6.64E-06	0.05. 0.12
I3D vs Doctor	0.91 vs 0.60	0.05	5.74	9.29E-09	0.20, 0.42
External test 1					
I3D vs Resnet 50	0.87 vs 0.71	0.03	6.57	5.08E-11	0.11, 0.21
I3D vs GBM	0.87 vs 0.80	0.02	4.49	7.11E-06	0.04, 0.11
I3D vs Doctor	0.87 vs 0.61	0.05	4.72	2.35E-06	0.15, 0.36
External test 2					
I3D vs Resnet 50	0.86 vs 0.71	0.03	5.83	5.59E-09	0.10, 0.20
I3D vs SVM	0.86 vs 0.66	0.02	9.2	3.62E-20	0.16, 0.24
I3D vs Doctor	0.86 vs 0.61	0.07	3.83	1.30E-04	0.12, 0.38

Note: The AUCs of I3D model was compared with other models in test sets, the P values were less than 0.05. The difference was statistically significant.

Table E2 Performance of Models for Classification of PCa in the Test Sets

Sets and Models	Sensitivity [95%CI](%)	Specificity [95%CI] (%)	Accuracy [95%CI] (%)	F1 score	AUC	P	kappa
Internal test							
XGB	64[49, 78]	75[60, 86]	70[60, 78]	.67	.74	<.01	.40
GBM	82[67, 92]	71[56, 83]	76[67, 84]	.77	.79	<.01	.53
SVM	80[65, 90]	75[60, 86]	77[68, 85]	.77	.82	<.01	.55
RF	73[58, 85]	81[67, 91]	77[68 85]	.76	.78	<.01	.55
ResNet50	47[32, 62]	**94[82, 98]**	71[61, 79]	.61	.75	<.01	.41
I3d	**91[78, 97]**	85[72, 94]	**88[80, 93]**	**.88**	**.91**	<.01	**.76**
Doctors	71[55, 83]	48[34, 63]	59[49, 69]		.60	.06*	.19
External test 1							
XGB	71[56, 83]	77[62, 86]	74[64, 82]	.73	.76	<.01	.48
GBM	92[79, 97]	56[41, 70]	74[64, 82]	.78	.80	<.01	.48
SVM	71[56, 83]	67[51, 79]	69[59, 77]	.69	.74	<.01	.38
RF	77[62, 88]	71[56, 83]	74[64, 82]	.75	.77	<.01	.48
ResNet50	52[37, 67]	81[67, 91]	67[57, 75]	.61	.71	<.01	.33
I3d	**83[69, 92]**	**83[69, 92]**	**83[69, 92]**	**.83**	**.87**	<.01	**.67**
Doctors	67[51, 79]	56[41, 70]	61[51, 71]		.61	.02	.23
External test 2							
XGB	61[45, 75]	64[45, 79]	62[51, 72]	.64	.59	.04	.24
GBM	51[35, 67]	**88[71, 96]**	68[56, 77]	.64	.66	<.01	.37
SVM	56[40, 71]	73[54, 86]	64[52, 74]	.63	.66	.01	.28
RF	66[49, 79]	64[45, 79]	65[54, 75]	.68	.65	.01	.29
ResNet50	63[47, 77]	76[57, 88]	69[58, 78]	.69	.71	<.01	.38
I3d	**81[65, 91]**	82[64, 92]	**81[71, 88]**	**.83**	**.86**	<.01	**.62**
Doctors	61[45, 75]	61[42, 77]	61[49, 71]		.61	.07*	.21

Note: Among machine learning models, SVM performs best in classification **of PCa in internal test set; GBM** performs best in external test set 1 and external test set 2.

Table E3 Details of I3D model prediction for prostate cancer with different Gleason's scores

	Maximum Gleason score	9	8	7	6
External test set 1	Cases	6	13	18	11
	I3D 1	5	10	15	10
	I3D 0	1	3	3	1
	Sensitive	83.30%	76.90%	83.30%	90.90%
External test set 2	Cases	3	16	10	12
	I3D 1	3	13	8	9
	I3D 0	0	3	2	3
	Sensitive	100%	81.30%	80%	75%

Note: I3D 0 represents the number of cases predicted to be benign by the I3D model; I3D 1 represents the number of cases predicted to be malignant by the I3D model. Because there was no negative control group, only sensitivity could be calculated.

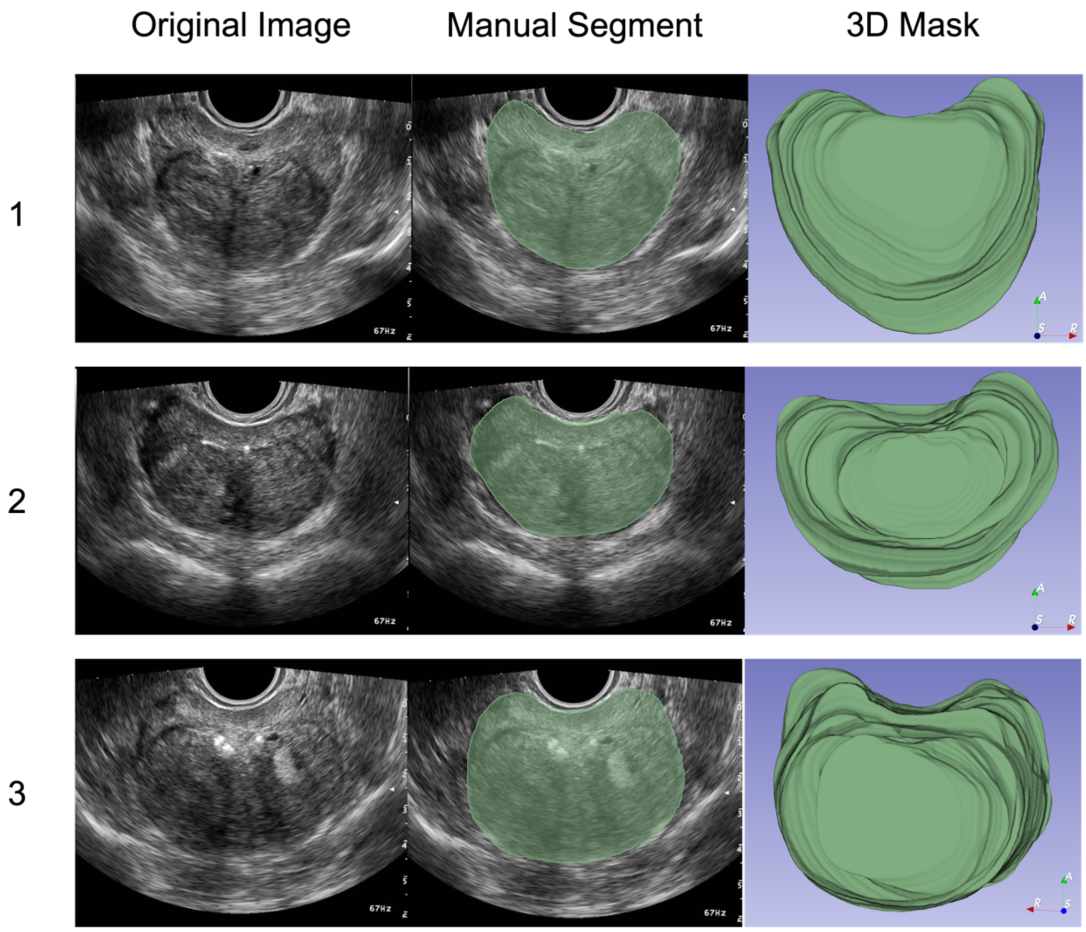

Figure E1 The examples of manual segmentation. (1) a 71-year-old man with prostate-specific antigen of 12.27 ng/mL and the result of biopsy pathology was Gleason grade group 6, the video clips was manual segmented by K.W. (2) a 71-year-old man with prostate-specific antigen of 4.23 ng/mL and the result of biopsy pathology was benign prostatic hyperplasia, the video clips was manual segmented by Q.H.L. (3) a 78-year-old man with prostate-specific antigen of 9.92 ng/mL and the result of biopsy pathology was Gleason grade group 7, the video clips was manual segmented by M.L.Z.

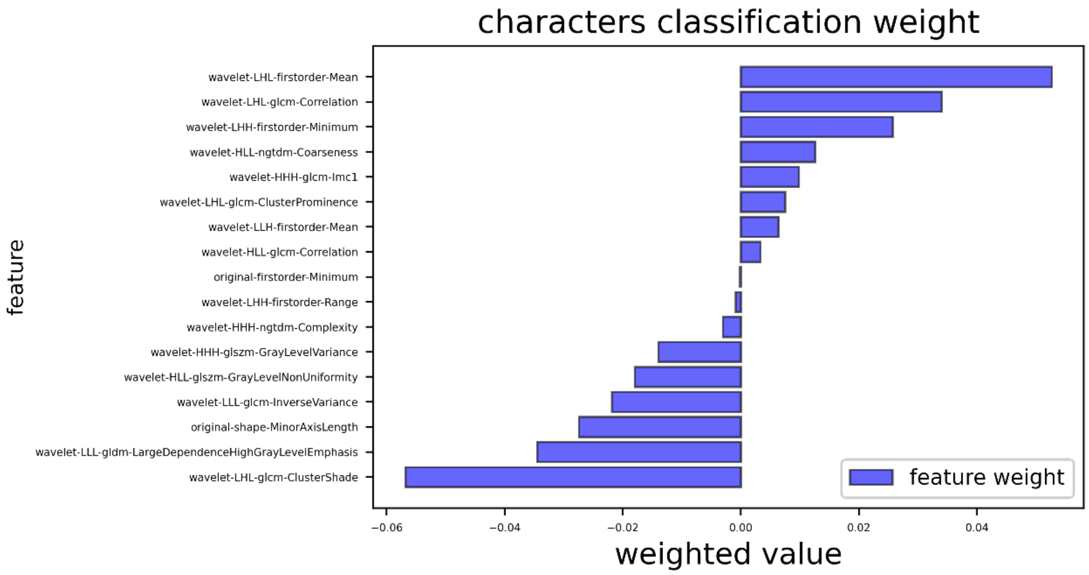

Figure E2 Characters classification weight of the ML features.

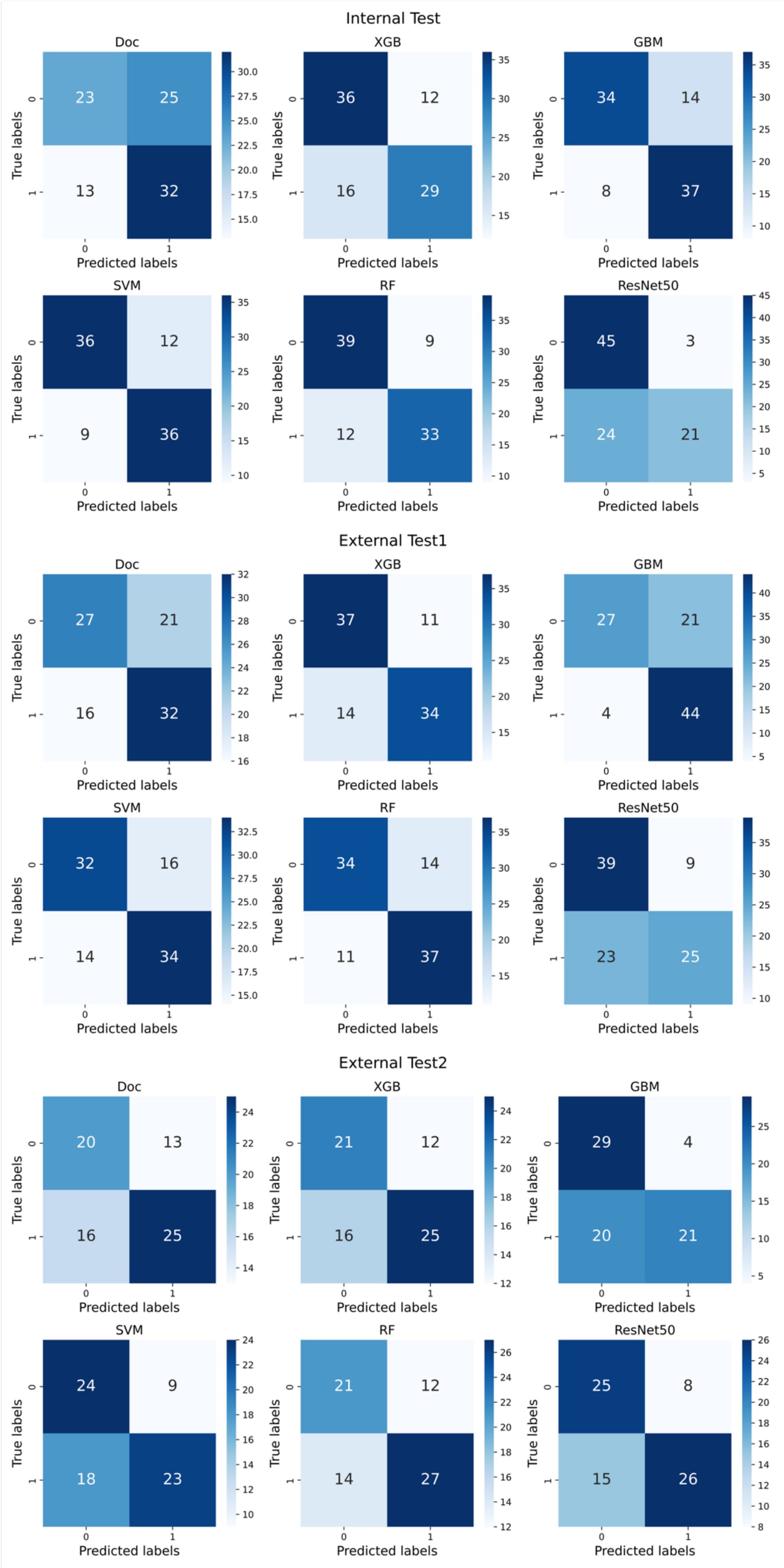

Figure E3 The confusion matrix used for other models

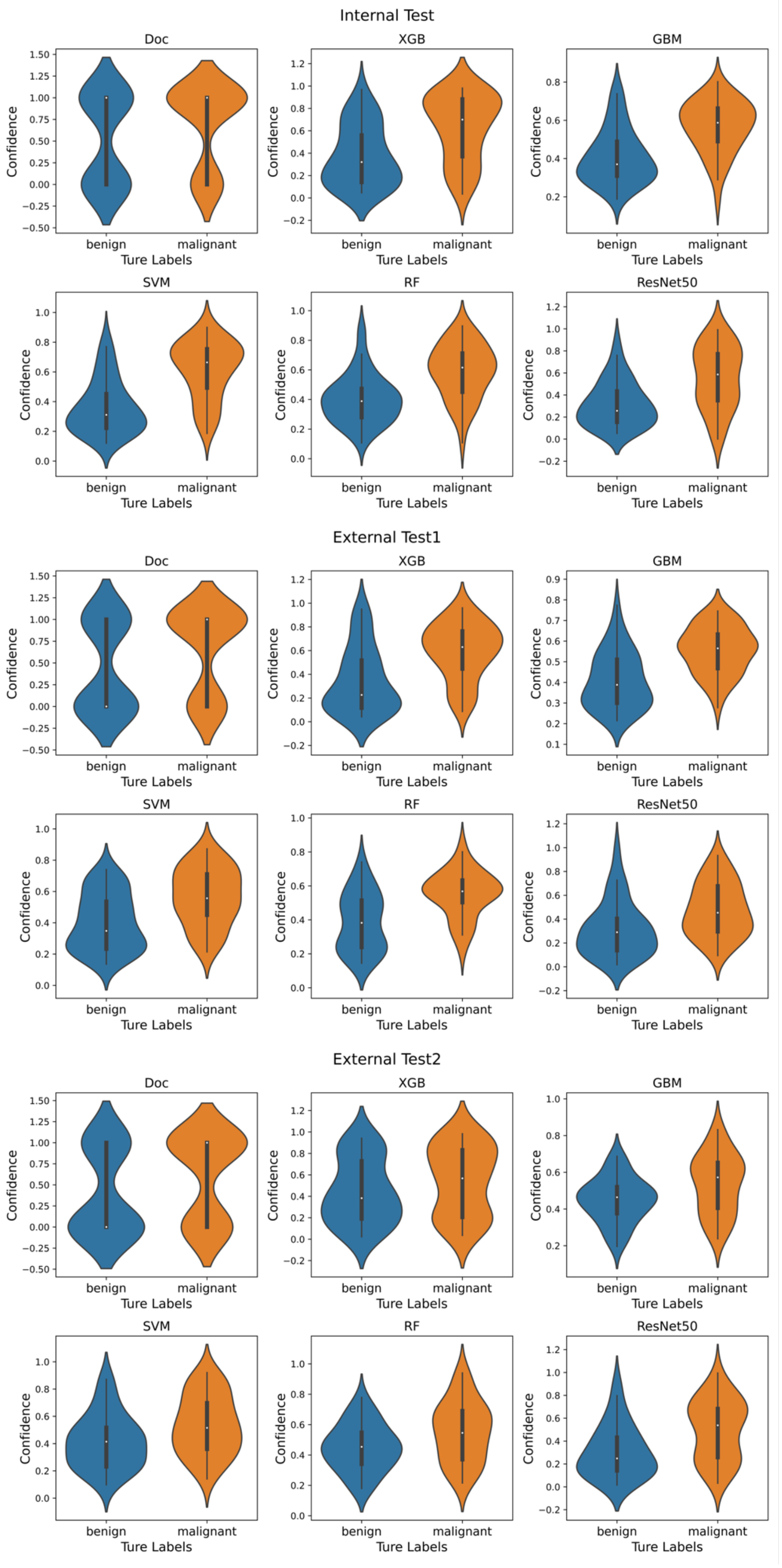

Figure E4 The violin plot used for other models
